# The gold-standard treatment for social anxiety disorder: A roadmap for the future

**DOI:** 10.3389/fpsyg.2022.1070975

**Published:** 2023-01-18

**Authors:** Nayeefa Chowdhury, Ahsan H. Khandoker

**Affiliations:** ^1^School of Psychological Sciences, Faculty of Medicine, Nursing and Health Science, Monash University, Melbourne, VIC, Australia; ^2^Healthcare Engineering Innovation Center, Department of Biomedical Engineering, Khalifa University, Abu Dhabi, United Arab Emirates

**Keywords:** social anxiety disorder, classical conditioning, randomized controlled trial, virtual reality, cognitive behavioral therapy, third-wave CBT, operant conditioning, gold-standard treatment

## Abstract

Exposure therapy (ET), which follows the Pavlovian extinction model, is regarded as the gold-standard treatment for social anxiety disorder (SAD). The prospect of virtual reality in lieu of a traditional laboratory setting for the treatment of SAD has not been rigorously explored. The aim of the review was to summarize, find gaps in the current literature, and formulate future research direction by identifying two broad research questions: the comparative efficacy between in vivo ET and virtual reality exposure therapy (VRET) and the effectiveness of the Pavlovian extinction model in treating SAD. The criteria for effectiveness were effect size, relapse prevention, attrition rate and ecological validity. A literature search on recent randomized controlled trials yielded a total of 6 original studies (N=358), excluding duplication and overlapping participants. All studies supported that VRET was as effective as in vivo ET. Behavioral therapy that follows classical conditioning principles has a high attrition and relapse rate. Comparisons were drawn between the efficacy of the Pavlovian extinction model and other existing models, including third-wave approaches. The neural markers are suggested to be included as efficacy measures in treating SAD. The gold-standard treatment for SAD requires a paradigm shift through rigorous longitudinal comparative studies.

## Introduction

Social anxiety disorder (SAD), a clinically diagnosed condition that leads to impairments in interpersonal settings due to fear of being judged (McKay and McKiernan, 2018), is the world’s sixth leading cause of disability (Baxter et al., 2014). The fear response is essential for survival, as it enables us to predict danger on cued signals and adapt to external environments (Krause and Domjan, 2017). However, the neural threat circuitry that enables us to regulate our emotions and behavior in changing environments is disrupted in a person with SAD (Kredlow et al., 2022). The development of social anxiety disorders could be explained by classical conditioning theory, attributed to Pavlov (Lilienfeld et al., 2019). Classical conditioning is an involuntary learning process of an association between two or more stimuli (Pavlov, 1897). A repetitive pairing of a neutral stimulus (e.g., people) with a biological stimulus (e.g., scream) elicits a conditioned response (e.g., avoidance) that was initially a reflex response to the biological stimulus (UCS) but is now displayed when the neutral stimulus presents alone (Pavlov, 1897). The benchmark treatments for SAD include exposure therapy (Steinman et al., 2016) and drug intervention (Klinger et al., 2005). Exposure therapy is an alternative to pharmacological interventions that produce undesirable side effects (Hindmarch, 2009). Exposure therapy (ET) follows the *extinction* principle (Pavlov, 1927) of classical conditioning, which repeatedly exposes the patients to the feared stimulus (CS) without the presence of UCS in a lab setting until the association between the UCS and CS is weakened, and the anxiety subsides (Hofmann, 2008). The digital revolution has brought about a change of modality from laboratory-based therapy to virtual reality exposure therapy (Bucci et al., 2019). Virtual reality is a 3D and 360-degree simulation of environments in which one can immerse and interact.

## Research questions and literature search

Can virtual reality treat social anxiety disorder using the principles of classical conditioning? The aim of this mini-review was to summarize, find gaps in the current literature, and formulate future research direction by identifying two broad research questions as follows:

- Is virtual reality exposure therapy (VRET) as effective as *in vivo* ET?
- Is ET based on the Pavlovian extinction model as effective as other ET or other therapy that use different principles or procedures in treating SAD?

**Figure 1:**
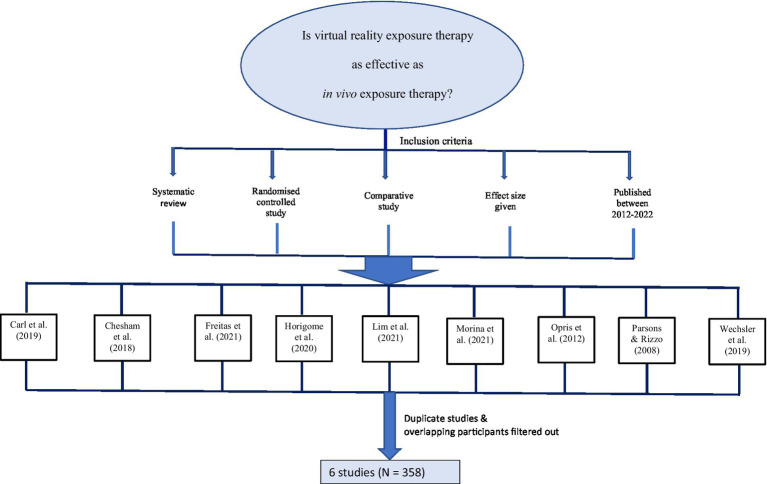
Literature selection process for the first research question.

The criteria of effectiveness were effect size, relapse prevention, attrition rate and ecological validity. A broader meta-analyses search was made in the scoping review to find the answer to the first research question (see Figure 1). Nine meta-analyses (Parsons and Rizzo, 2008; Opriş et al., 2012; Chesham et al., 2018; Carl et al., 2019; Wechsler et al., 2019; Horigome et al., 2020; Freitas et al., 2021; Morina et al., 2021; Lim et al., 2022), which included randomized control trials, provided effect sizes between the comparison groups, were published in the last decade and the English language were selected for the review. The meta-analyses covered substantially overlapping studies. Any duplication or studies with less than 10 participants in the experimental (i.e., SAD) group were excluded from this review. A total of 6 studies (*N* = 358) fitted the eligibility criteria. Table 1 represents all randomized controlled comparative studies on SAD, which were included in the seven meta-analyses. The control groups (*n*_C_ = 108) and participants in the VRET (*n*_VRET_ = 121) and *in vivo* ET (*n*_IVET_ = 129) studies in Table 1 were *unique* individuals. Overlapping participants, including follow-up studies, were excluded to avoid depicting a larger sample in a misleading way. For example, Safir et al. (2012) conducted a study with the same set of participants that initially participated in Wallach et al.’s (2009) study; hence, they were not duplicated in the table; and Anderson et al.’s (2017) follow-up study comprising the same group of original participants in a past study (Anderson et al., 2013) was excluded from the table. Two studies (Wallach et al., 2009; Anderson et al., 2013) listed in the table focused on fear of public speaking, which is a subset of SAD.

**Table 1:** Virtual reality exposure therapy (VRET) for the treatment of social anxiety disorder (SAD): randomized controlled comparative studies.

ID of the Studies	Comparison	Sample size				Total sample size	Cumulative sample size (*N*)	Effect size after treatment^*^ (Hedges’ *g*)
		VRET	IVET	WL		358	VRET vs. WL	VRET vs. IVET
Anderson et al., (2013)^a^	• VRET vs. IVGET • VRET vs. WL	25	25	25	75		0.76	–0.61
Bouchard et al. (2017)	• VRCBT vs. IVCBT • VRCBT vs. WL	17	22	20	59		1.53	0.56
Kampmann et al. (2016)^b^	• VRET vs. IVET • VRET vs. WL	19	18	18	55		0.61	–0.55
Klinger et al. (2005)	• VRCBT vs. IVGCBT	18	18	0	36		N/A	0.37
Robillard et al. (2010)	• VRCBT vs. IVCBT • VRCBT vs. WL	14	16	15	45		1.53	0.61
Wallach et al. (2009)	• VRCBT vs. IVCBT • VRCBT vs. WL	28	30	30	88		1.14	0.08
		= 121 (*n*_VRET_)	= 129 (*n*_IVET_)	= 108 (*n*_C_)				

*Note.* VRET = virtual reality exposure therapy; IVET = *in vivo* exposure therapy; IVGET = *in vivo* group exposure therapy; VRCBT = virtual reality cognitive behavioral therapy; IVCBT = *in vivo* cognitive behavioral therapy; IVGCBT = *in vivo* group cognitive behavioral therapy; WL = waiting-list; N/A = not applicable; *n*C = Total number of individuals in the control groups; ^*^Hedges’ g values were taken from Carl et al. (2019, p. 30); ^a^Some form of cognitive intervention was made; ^b^No cognitive intervention was made.

## Results

All studies, except for Kampmann et al.’s (2016) study (to be discussed later in this review), in Table 1 unequivocally suggest that the efficacy of VRET in treating SAD is clinically significant (i.e., Hedges’ *g* = 0.80–1.53) compared to control groups. Furthermore, none of them shows evidence that the effectiveness of VRET is inferior to *in vivo* ET. To date, only one longitudinal study has been done on the comparative efficacy between VRET and *in vivo* ET (Anderson et al., 2017). The 6-year longitudinal study (*N* = 28) that Anderson et al. (2017) investigated showed no difference between VRET and *in vivo* ET. The effect size that compared the self-rating scores between the two groups of participants, using the ‘fear of native evaluation’ (FNE) scale, was clinically non-significant (i.e., hedges’ *g* = −0.15). This finding favors VRET over *in vivo* ET from an ecological standpoint. Extinction trials in ET are required to be conducted in multiple contexts and settings to prevent a relapse of SAD (Vervliet et al., 2013). The delivery of *in vivo* ET in different settings and contexts is expensive. VRET serves as an ecologically valid option for treating SAD.

## Discussion

### VRET versus *in vivo* ET

Questions may arise about the intensity and frequency of VRET sessions being appropriate for patients with SAD. All VRET studies included in this literature review tailored the virtual environments according to the pace and fear hierarchy of each participant during the VR therapy sessions in the presence of professional therapists (Klinger et al., 2005; Wallach et al., 2009; Robillard et al., 2010; Anderson et al., 2013; Kampmann et al., 2016; Bouchard et al., 2017). The therapists could see the participant’s field of view in real-time and simultaneously observe his or her responses (Kampmann et al., 2016). All VRET sessions were conducted in lab settings to avoid confounding variables, as the *in-vivo* ET studies took place in lab settings (Klinger et al., 2005; Wallach et al., 2009; Robillard et al., 2010; Anderson et al., 2013; Kampmann et al., 2016; Bouchard et al., 2017). However, VRET sessions can also be conducted in the comfort of one’s home (Hartanto et al., 2015; Emmelkamp et al., 2020; Miloff et al., 2020; Stefaniak et al., 2022). Home-based, self-guided VRET yields an additional advantage over *in-vivo* ET in that some patients with severe SAD may find direct interaction with the therapist intimidating (Hartanto et al., 2015). Systematic studies are required, however, to investigate the transferability of skills acquired during home-based VRET sessions to real-life scenarios. One study found that the closer the VR environment mimicked the real-life environment in terms of the cultural setting and grooming of VR characters, the more effective the therapy was (Wallach et al., 2009).

### Attrition rate

The dropout rate is another critical indicator of the efficacy of a treatment. Two studies show that the attrition rate from *in vivo* ET was more than twice as much as that from the VRET (Safir et al., 2012; Anderson et al., 2013), suggesting VRET’s efficacy over *in vivo* ET for the treatment of SAD. Nonetheless, a 9–35% attrition rate (Bentley et al., 2021) calls the effectiveness of ET that employs the Pavlovian extinction model into question. Additionally, classical conditioning fails to prevent relapse in SAD patients (Pavlov, 1897; Pittig et al., 2018; Levy et al., 2022).

### Relapse prevention

Relapse prevention is a predictive marker of efficacy. A recent study shows that 21.8% of SAD patients relapse after achieving successful extinction through Pavlovian ET (Scholten et al., 2021). There is a knowledge gap on the efficacy of classical conditioning in relapse prevention due to a dearth of longitudinal studies. One 3-month follow-up study (Mattick et al., 1989) revealed the superiority of cognitive restructuring over the sole Pavlovian extinction model. Likewise, Ougrin’s (2011) meta-analysis showed that the superiority of cognitive intervention strategies over the Pavlovian extinction model was statistically significant at follow-up (6–12 months). The findings are consistent with the findings of Kampmann et al.’s (2016) study (see Table 1) that exclusively administered behavioral therapy and excluded cognitive counterparts from exposure therapy.

In contrast to all VRET studies listed in Table 1, Kampmann et al. (2016) attempted to investigate the sole effects of the Pavlovian extinction model in treating SAD. Compared to the control group, the effect size was not clinically significant (see Table 1). The findings underscore the possible inadequacy of the Pavlovian extinction model in treating SAD. Do other therapies that are not rooted in Pavlovian classical conditioning fare better in relapse prevention in SAD patients?

### Alternative approaches

Acceptance and commitment therapy (ACT) uses the principles of operant conditioning and attempts to shift the focus of SAD patients to holistic well-being from the feared stimulus (Toghiani et al., 2019). Further investigations are needed to compare extinction strategies rooted in operant conditioning with the Pavlovian classical conditioning model. ET strategies that tweaked or deviated from the Pavlovian principles of classical conditioning during the process of extinction show more effectiveness in relapse prevention (Craske et al., 2018). Three studies demonstrated that occasional exposure to UCS, either paired or unpaired with CS during extinction trials, helps prevent relapse compared to Pavlovian classical conditioning (Bouton, 2004; Thompson et al., 2018; Lipp et al., 2021). Craske et al. (2014, p. 11) suggested that during “extinction trials,” a new “inhibitory” learning takes place instead of an unlearning of the previous association. This is supported by Shin and Liberzon’s (2010) study on neurocircuitry during fear conditioning and extinction. The recent advancement in neuroscience has made it possible to pinpoint neural markers of SAD.

Several studies reveal that the difference between the brain activity of the prefrontal cortex of SAD and control groups at baseline is statistically significant (Pittig et al., 2018; Lee et al., 2021; Kredlow et al., 2022). There is a dearth of research pertaining to comparative studies on self-reported scores and neural correlates during VRET in treating SAD. A recent VRET study showed that self-rating scores were consistent with neural correlates in SAD (Lee et al., 2021).

## Concluding remarks

To conclude, recent studies provide strong evidence that VRET is as effective as *in vivo* ET in treating SAD. VRET has higher ecological validity than *in vivo* ET. Self-guided VRET in a home setting requires rigorous future investigations on the feasibility of data-driven mechanisms through remote or automated monitoring that ensure optimal intervention and prevent burnout. The future research direction of VRET studies in treating SAD should be geared toward investigating the relationship between the cultural paradigm of the VR environment and the extent of generalization of skills from virtual to real-life environments, and the relevance of designing culturally sensitive VR software. SAD treatment strategies based on classical conditioning have high attrition and relapse rate. There is a gap in the literature estimating the efficacy of therapies based on attrition rate and relapse prevention. Future research should be geared toward comparative longitudinal, relapse-prevention studies between Pavlovian exposure therapy, cognitive therapy, and third-wave approaches, such as therapy based on operant conditioning, and include both self-rating scales and neural markers as efficacy measures in treating SAD.

## Author contributions

NC conceived the idea, designed the review protocol, conducted the literature search and drafted the article. AK revised the article and organized the funding. All authors approved the submitted version.

## Funding

This work was supported by three grants [8474000221; KKJRC- 2019-Health2 (Khandoker), CIRA 2021-051 (8474000408), and HEIC 8474000132] from Khalifa University Abu Dhabi, UAE.

## Conflict of interest

The authors declare that the research was conducted in the absence of any commercial or financial relationships that could be construed as a potential conflict of interest.

The reviewer MA declared a past co-authorship with the author AK to the handling editor.

## Publisher’s note

All claims expressed in this article are solely those of the authors and do not necessarily represent those of their affiliated organizations, or those of the publisher, the editors and the reviewers. Any product that may be evaluated in this article, or claim that may be made by its manufacturer, is not guaranteed or endorsed by the publisher.
